# Supplementary material for: Serum microRNAs as potential new biomarkers for cisplatin resistance in gastric cancer patients
Source: PeerJ. 2020 Apr 14;8:e8943. doi: 10.7717/peerj.8943 (PMC7164432; doi:10.7717/peerj.8943)
Supplement: Table S1 [file peerj-08-8943-s001.docx]

| **The common target genes among four different databases of the 7 miRNAs shown in Figure 3.** | | | | | | | | | |
| --- | --- | --- | --- | --- | --- | --- | --- | --- | --- |
| miR-9-3p | miR-9-5p | | | | miR-146a-5p | miR-370-3p | miR-433-3p | miR-519a-5p | miR-522-5p |
| ACTBL2 | ETS1 | PNRC2 | FERMT1 | ZBED3 | COPS8 | RETSAT | CHD9 | CBX4 | BEND4 |
| KIAA0408 | COL12A1 | MTHFD2 | POU2F2 | ATXN7 | CARD10 | PRPF38A | KRAS | TEF | TEF |
| PCMTD1 | GPBP1L1 | LMNA | UBE4B | DICER1 | ZNF117 | NF1 | COX6B1 | SLC31A1 | ATP11A |
| CREBRF | EOGT | PI4K2A | SERINC5 | CD34 | GPM6B | PARVB | NINJ1 | TJAP1 | CBX4 |
| IGF2 | AUH | BACE1 | SYAP1 | KIF1C | IFIT3 | TGFBR2 | MAFK | CDC5L | SLC31A1 |
| CRK | ZNF557 | TBPL1 | TGFBR2 | PTPRK | TRAF6 | RAB7A | MRPS25 | BEND4 | CDC5L |
| MTRNR2L6 | ESR1 | KCNJ2 | SLC39A14 | CHSY1 | ARL8A | KCTD15 | HIVEP1 | ATP11A | TJAP1 |
| SEC24A | BCL6 | SEC23IP | DRD2 | NCOR2 | RHOBTB3 | RAD54L2 | WDR45B |  |  |
| ITPRIPL2 | ATP7A | IGF2R | REST | MYLK | CCDC6 | CANX | AZIN1 |  |  |
| YOD1 | CCNDBP1 | PPP4R2 | MAP1B | PRRX1 | METTL7A | BAG4 | MAPK8 |  |  |
| RCOR1 | TGFBI | FBN2 | PXDN | CPEB4 | ELAVL1 | HSP90AB1 | LRIG3 |  |  |
| TPD52 | PPARA | PRDM1 | OPTN | ELAVL1 | RAC1 | DHX33 | BRWD1 |  |  |
| KLHL15 | LHFPL2 | SOCS5 | ATP11C | HIST1H2AE | IRAK1 | MGMT | GRB2 |  |  |
| REST | FOXO1 | ID4 | SIRT1 | CPA4 | PTGS2 | LIN28A | UGT2B4 |  |  |
| ITGB1 | EN2 | AMOTL1 | BCL2L11 | SLC19A3 | AVL9 | ARID3B | STK38 |  |  |
| FBXO28 | KLF5 | AP3B1 | STK3 | MDM4 | UMPS | FOXO1 |  |  |  |
| ELOVL5 | RAB34 | CAPZA1 | NR2E1 | EDEM3 | NUMB | KIAA0355 |  |  |  |
| ZNF148 | COLEC12 | MAP3K3 | MYH9 | PRKD3 | CFH | HMGA2 |  |  |  |
| GXYLT1 | SLC35B3 | CCNG1 | FLNB | STMN1 | ROBO1 | TNRC6B |  |  |  |
| SLC38A2 | SDC1 | P4HA2 | CXCR4 | FOXP1 | PPP1R11 | CYB561D1 |  |  |  |
| CAPZA1 | CHMP2B | SYNGR2 | ZNF407 | TC2N | SHCBP1 | NFASC |  |  |  |
| NHS | SNX7 | POU2F1 | ONECUT2 | SPAG9 | ERBB4 | DCK |  |  |  |
| GPR180 | TRPM7 | ANP32B | ZFAND1 | PRTG | PRKCE |  |  |  |  |
| B4GALT6 | NFATC3 | EFNA1 | TESK2 | FOXO3 | CCL5 |  |  |  |  |
| PKD2L2 | RYBP | PIGM | FNBP1 | TRIM14 | CCR9 |  |  |  |  |
|  | LDLRAP1 | SRF | UHMK1 | PCMTD2 | PMAIP1 |  |  |  |  |
